# Epithelial microRNA-30a-3p targets RUNX2/HMGB1 axis to suppress airway eosinophilic inflammation in asthma

**DOI:** 10.1186/s12931-022-01933-x

**Published:** 2022-01-29

**Authors:** Wenliang Wu, Jiali Gao, Dian Chen, Gongqi Chen, Yuchen Feng, Chenli Chang, Shengchong Chen, Lingling Yi, Guohua Zhen

**Affiliations:** 1 Division of Respiratory and Critical Care Medicine, Department of Internal Medicine, Tongji Hospital, Tongji Medical College, Huazhong University of Science and Technology, Wuhan, 430030 China; 2 Key Laboratory of Respiratory Diseases, National Health Commission of People’s Republic of China, and National Clinical Research Center for Respiratory Diseases, Wuhan, China

**Keywords:** Epithelial cells, miR-30a-3p, Eosinophilia, RUNX2, HMGB1, Asthma

## Abstract

**Background:**

Type 2-high asthma is a prominent endotype of asthma which is characterized by airway eosinophilic inflammation. Airway epithelial cells play a critical role in the pathogenesis of asthma. Our previous miRNA profiling data showed that miR-30a-3p was downregulated in bronchial epithelial cells from asthma patients. We hypothesize that epithelial miR-30a-3p plays a role in asthma airway inflammation.

**Methods:**

We measured miR‐30a-3p expression in bronchial brushings of asthma patients (n = 51) and healthy controls (n = 16), and analyzed the correlations between miR‐30a-3p expression and airway eosinophilia. We examined whether Runt-related transcription factor 2 (*RUNX2*) was a target of miR‐30a-3p and whether RUNX2 bound to the promoter of high mobility group box 1 (*HMGB1*) by using luciferase reporter assay and chromatin immunoprecipitation (ChIP)-PCR. The role of miR‐30a-3p was also investigated in a murine model of allergic airway inflammation.

**Results:**

We found that miR-30a-3p expression were significantly decreased in bronchial brushings of asthma patients compared to control subjects. Epithelial miR-30a-3p expression was negatively correlated with parameters reflecting airway eosinophilia including eosinophils in induced sputum and bronchial biopsies, and fraction of exhaled nitric oxide in asthma patients. We verified that *RUNX2* is a target of miR-30a-3p. Furthermore, RUNX2 bound to the promoter of *HMGB1* and upregulated HMGB1 expression. RUNX2 and HMGB1 expression was both enhanced in airway epithelium and was correlated with each other in asthma patients. Inhibition of miR-30a-3p enhanced RUNX2 and HMGB1 expression, and RUNX2 overexpression upregulated HMGB1 in BEAS-2B cells. Intriguingly, airway overexpression of mmu-miR-30a-3p suppressed Runx2 and Hmgb1 expression, and alleviated airway eosinophilia in a mouse model of allergic airway inflammation.

**Conclusions:**

Epithelial miR-30a-3p could possibly target RUNX2/HMGB1 axis to suppress airway eosinophilia in asthma.

**Supplementary Information:**

The online version contains supplementary material available at 10.1186/s12931-022-01933-x.

## Background

Asthma is a chronic airway disease characterized by airway hyperresponsiveness (AHR), airway inflammation, mucus overproduction, and submucosal fibrosis [1]. Type 2 immune response driven by the type 2 cytokines, IL-4, IL-5, and IL-13, plays an important role in the pathogenesis of asthma. The type 2-high and type 2-low asthma endotypes are classified according to the expression levels of the type 2 cytokines [2]. Airway eosinophilic inflammation is a key feature of type 2-high asthma [3–6]. Airway epithelial cells contribute to airway eosinophilia by producing chemokines including C–C motif chemokine ligand (CCL)11, CCL24, and CCL26.

MicroRNAs (miRNAs) are ~ 22 nucleotide long, non-coding RNAs that play a critical role in the regulation of gene expression. MiRNAs have been implicated in the pathogenesis of asthma [7–9]. Significant alterations in miRNA expression of airway epithelial cells have been reported by us and others [10–12]. A recent study demonstrated that epithelial miR-141 regulates airway mucus production in asthma [7]. We previously reported that epithelial miRNAs were involved in asthmatic airway eosinophilia [10–12]. Our miRNA profiling data showed that a set of epithelial miRNAs including miR-30a-3p was downregulated in asthma patients [12]. MiR-30a-3p was reported to regulate the proliferation and apoptosis of cancer cells [13, 14]. A recent study showed that miR-30a-3p expression was significantly decreased in the peripheral blood of asthmatic patients [15]. However, the role of airway epithelial miR-30a-3p in the pathogenesis of asthma remains unclear. We hypothesized that epithelial miR-30a-3p is involved in airway eosinophilic inflammation in asthma.

Runt-related transcription factors (RUNXs) serve as key regulators in development and carcinogenesis [16–18]. There are three *RUNX* genes, *RUNX1*, *RUNX2* and *RUNX3*, in mammals. Maternal smoking could promote the development of asthma in the offspring by upregulating the expression of RUNX1 [19]. RUNX2 is reported to promote the gene transcription of SAM pointed domain containing ETS transcription factor (*SPDEF*), a key transcription factor in goblet cell differentiation [20]. A previous study suggests that RUNX2 may bind to the promoter of high mobility group box 1 (*HMGB1*) [21].

HMGB1 is a pro-inflammatory mediator belonging to the alarmin family [22]. HMGB1 can interact with multiple surface receptors including toll-like receptors to promote inflammatory response [23]. It was reported that HMGB1 enhanced the survival of eosinophils and served as a chemoattractant for eosinophils [24, 25]. HMGB1 levels in induced sputum from asthmatic patients were elevated [26]. In animal models of asthma, airway eosinophilic inflammation was ameliorated by blocking HMGB1 activity [26, 27]. These studies indicate that HMGB1 contributes to airway eosinophilia in asthma.

In the present study, we found that downregulated epithelial miR-30a-3p expression was negatively correlated with airway eosinophilic inflammation in asthma patients. *RUNX2* was a target of miR-30a-3p, and RUNX2 directly regulated HMGB1 expression by binding to the promoter of *HMGB1*. Epithelial RUNX2 and HMGB1 expression was both enhanced in asthma patients. In vitro, inhibition of miR-30a-3p expression promoted IL-13-induced RUNX2 and HMGB1 expression in airway epithelial cells. Intriguingly, airway overexpression of mmu-miR-30a-3p alleviated airway eosinophilia in a mouse model of allergic airway inflammation.

## Materials and methods

### Subjects

We recruited 16 control subjects and 51 patients with confirmed asthma who have obvious symptoms and. All subjects were recruited from Tongji Hospital. Asthma were diagnosed by a physician; had symptoms of episodic cough, wheeze and/or dyspnea; and had accumulated dosage of methacholine provoking a 20% fall (PD_20_) of forced expiratory volume in the first second (FEV_1_) < 2.505 mg and/or ≥ 12% increase in FEV_1_ following inhalation of 200 μg salbutamol. Control subjects had no respiratory symptoms, normal spirometry value and methacholine PD_20_ ≥ 2.505 mg. None of the subjects had ever smoked or received inhaled or oral corticosteroid or leukotriene antagonist. Written informed consents were obtained from all subjects. The ethics committee of Tongji Hospital, Huazhong University of Science and Technology had approved the study.

For each subject, we recorded their demographic information, collected sputum specimens, and measured spirometry and fraction of exhaled nitric oxide (FeNO) at study entry. Bronchoscopy with brushing and endobronchial biopsies was performed within 1 week of study entry. Methods for histology and biopsy techniques, pulmonary function testing and FeNO measurement were described previously [28].

### Murine model of allergic airway inflammation

Six‐ to eight‐week‐old female C57BL/6J mice were sensitized with intraperitoneal injection of OVA solution (100 μg in 100 μL saline, Sigma‐Aldrich, USA) mixed with Al(OH)_3_ as an adjuvant on days 0, 7 and 14. Mice were challenged with intranasal administration of OVA solution (1 mg in 50 μL saline) on days 21, 22, 23, 24 and 25. Mmu‐miR‐30a‐3p agomir (5 nmol in 40 μL saline, RiboBio, China) or control agomir was administered intranasally 2 h before OVA challenge on days 21 and 23. Mice were sacrificed 24 h after the last OVA challenge. Lung tissues were collected for histological analysis and quantitative PCR. Animal experiments were approved by the ethics committee of Tongji Hospital, Huazhong University of Science and Technology.

### Assessment of airway inflammation

Cell counts for macrophages, eosinophils, lymphocytes, and neutrophils in bronchoalveolar lavage fluid (BALF) were performed. The severity of peribronchial inflammation in H&E-stained mouse lung sections was scored using the following features: 0, normal; 1, few cells; 2, a ring of inflammatory cells (1 cell layer deep); 3, a ring of inflammatory cells (2–4 cells deep); 4, a ring of inflammatory cells (> 4 cells deep) [29].

### Histology, immunohistochemistry and periodic acid-Schiff (PAS) staining

The human airway biopsy and mouse left lungs were paraffin-embedded, and 5-μm sections were cut. Sections were used for hematoxylin and eosin (H&E) staining, periodic acid-Schiff (PAS) staining and immunohistochemical examination. For immunofluorescence staining, bronchial biopsy sections underwent antigen retrieval and were incubated with mouse monoclonal RUNX2 antibody (sc-390715 X, 1:1000 dilution, Santa Cruz Biotechnology, USA) and rabbit polyclonal HMGB1 antibody (10829-1-AP, 1:200 dilution, proteintech, China), then incubated with Cy3 goat anti-mouse secondary antibody (ServiceBio, China) and FITC goat anti-rabbit secondary antibody (ServiceBio, China). Nuclei were stained with DAPI. Photographs were taken by using a fluorescence microscope (NIKON Eclipse ci, Japan). For PAS staining, lung sections were stained with PAS (Goodbio technology, China) for detection of mucus. The number of PAS-staining-positive cells was counted in 10 random fields for each lung section at ×200 magnification. Observers who were blinded to the clinical status of the subjects counted numbers of eosinophils/mm^2^ submucosa as previously described [28].

### Cell culture and treatment

BEAS‐2B cell lines were purchased from ATCC (Manassas, VA). Cells were cultured in DMEM medium (Gibco, USA) with 10% FBS (Biological Industries, Israel). Cells were stimulated with or without IL-13 (20 ng/mL, Peprotech, USA) and transfected with control or miR-30a-3p mimic (50 μmol/L, RiboBio, China), control or miR-30a-3p inhibitor (100 μmol/L, RiboBio, China), scrambled or RUNX2 siRNA (100 μmol/L, RiboBio, China), and empty control or RUNX2 cDNA expression vector (500 ng/mL, GeneCopoeia, China) using Lipofectamine 3000 (Invitrogen, USA). The sequence of the sense strand of RUNX2 siRNA (5′-CTCTGCACCAAGTCCTTTTdTT‐3′) have been described previously [21]. Forty-eight hours after IL-13 stimulation, cells were harvested for quantitative PCR and Western blotting. Cell culture medium was collected for ELISA.

### Quantitative PCR

Total RNA from human bronchial brushings, mouse lungs and BEAS-2B cells was isolated using TRIzol (Invitrogen, USA) and reversely transcribed to cDNA using PrimeScript RT reagent kit (Takara, Japan). The sequences of the primers for Sybr Green real-time PCR were obtained from PrimerBank. Other primer sequences were designed by Sangon Biotech, China. The transcript levels of 5 s, miR-30a-3p, *GUSB*, *RUNX2*, *HMGB1*, *Gapdh*, *Runx2*, *Hmgb1* and *Muc5ac* were determined by using Takara SYBR Premix ExTaq polymerase and a CFX Connect PCR System (Bio-Rad Laboratories, USA). Fold differences were determined by the 2^−ΔΔCT^ method [30]. The gene expression was expressed as log2 transformed and relative to the median of control subjects or the mean of control group. The primers used are listed in Additional file 1: Table S2.

### *Fluorescence *in situ* hybridization*

We performed fluorescence in situ hybridization of hsa- and mmu-miR-30a-3p on paraffin-embedded sections using digoxin-labeled hsa- and mmu-miR-30a-3p miRNA probe (Qiagen, China). The sequence of the probes for both hsa-and mmu-miR-30a-3p was 5′- GCTGCAAACATCCGACTGAAAG -3′. The protocol was according to the manufacturer’s instructions.

### Chromatin immunoprecipitation (ChIP) assay

ChIP assays were performed using a chromatin immunoprecipitation (ChIP) kit (#56383, Cell Signaling Technology, USA). BEAS-2B were treated with IL-13 (20 ng/mL, Biolegend, USA) for 48 h. Chromatin was sonicated to obtain 100–900-bp DNA/protein fragments after cells were cross-linked with formaldehyde. Then, anti-RUNX2 (rabbit mAb, D1L7F, Cell Signaling Technology, USA) or IgG were added to lysates and incubated at 4 °C overnight. The next day, ChIP-Grade Protein G Magnetic Beads were used for immunoprecipitation. Then DNA was released and purified for subsequent PCR and agarose gel electrophoresis. Standard PCR were performed to amplify human *HMGB1* promoter region containing the AAACCACAG sequence using the primers 5′- CGTGGTCTGCTCAGGCTAAA -3′ (forward) and 5′- GCATGTGCCCAAATCCACAG -3′ (reverse).

### Luciferase activity assay

The vectors (pEZX-MT06, GeneCopoeia, USA) harboring wild-type, mutant *RUNX2* 3′-UTR or no 3′-UTR (control) were co-transfected with miR-30a-3p mimic or non-targeting control miRNA in BEAS-2B cells. After 24 h, cells were harvested and lysed and luciferase activity was measured with a Dual-Luciferase Reporter Assay Kit (Promega, USA). The firefly luciferase activity was normalized to renilla luciferase activity. To investigate whether RUNX2 binds to the promoter of *HMGB1*, the wild type, truncated, or mutant *HMGB1* promoters were cloned into a pPro-RB-Report vector (RiboBio, China). These luciferase vectors were co-transfected with empty control or RUNX2 cDNA expression vector in BEAS-2B cells. After 24 h, cells were harvested and luciferase activity was measured with a Dual-Luciferase Reporter Assay Kit (Promega, USA). The renilla luciferase activity was normalized to firefly luciferase activity.

### Western blotting

Proteins were extracted from BEAS-2B cells using RIPA buffer (Servicebio, China). Fifty micrograms of extracted proteins were separated using 10% SDS-PAGE, and the separated proteins were transferred onto polyvinylidene difluoride (PVDF) membranes (Roche, Germany). The membranes were first probed with indicated primary antibodies. Antibodies used in Western blot were: RUNX2 (Abcam, ab23981, 1:1000 dilution), HMGB1 (Proteintech, 10829-1-AP, 1:2000 dilution), GAPDH (Aspen, 1:2000 dilution). Then antibodies were detected using horseradish peroxidase-conjugated goat anti-rabbit IgG (Aspen, 1:4000 dilution) secondary antibody followed by ECL Western blot detection reagent (MedChemExpress, USA). Densitometry was assessed using ImageJ (National Institutes of Health, USA) and normalized to GAPDH.

### Enzyme linked immunosorbent assay (ELISA)

HMGB1 protein levels in supernatant from BEAS-2B cell culture media and BALF of mouse was analyzed by ELISA (Shino-Test Corporation, Japan). ELISA was performed according to the manufacturer's instructions. All samples and standards were measured in duplicate.

### Statistical analysis

We analyzed data using Prism version 7.3 (GraphPad Software, San Diego, CA, USA). For normally distributed data, we calculated the means ± standard deviation (SD) and used parametric tests (unpaired Student’s *t* test or one-way analysis of variance with Tukey correction) to compare across groups. For non-normally distributed data, we calculated medians (with interquartile ranges) and used non-parametric tests (Mann–Whitney test or Kruskal–Wallis test with Dunn intergroup comparison). We analyzed correlations using Spearman’s rank order correlation. Values of *P* < 0.05 were considered statistically significant.

## Results

### Epithelial miR-30a-3p expression is decreased and associates with airway eosinophilic inflammation in asthma

**Fig. 1:**
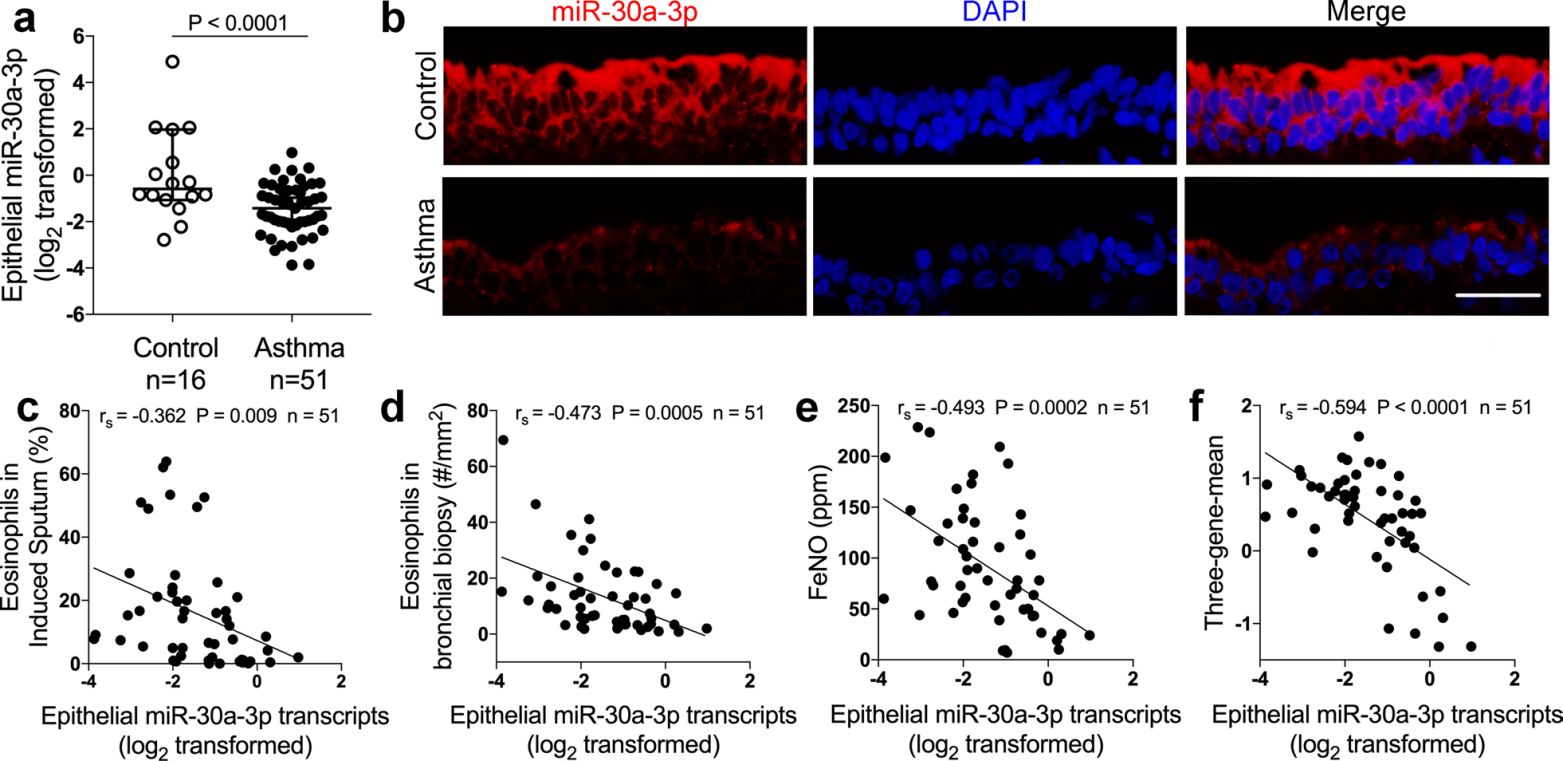
Epithelial miR-30a-3p expression is decreased and associates with airway eosinophilic inflammation in asthma. **a** Bronchial brushings from asthma patients (n = 51) and control subjects (n = 16) were subjected to quantitative PCR assays of hsa-miR-30a-3p transcript levels. The transcript levels were expressed as log2 transformed and relative to the median value for controls (two‐tailed Mann–Whitney test). **b** Representative images of fluorescence in situ hybridization of miR‐30a‐3p in epithelium of bronchial biopsies from asthma patients and controls. Scale bar, 50 μm. **c**–**f** Correlation assays between epithelial miR‐30a-3p transcript levels and eosinophils in induced sputum (**c**) and bronchial biopsies (**d**), FeNO (**e**), and three‐gene‐mean of *CLCA1*, *POSTN* and *SERPINB2* (**f**) in asthma patients (n = 51). Correlation assays were performed using Spearman’s rank‐order correlation

Subject characteristics are summarized in Table 1. We measured the expression of miR-30a-3p in endobronchial brushing samples from treatment‐naïve asthma patients (n = 51) compared to control subjects (n = 16) using quantitative PCR. We found that miR‐30a‐3p expression was significantly decreased in bronchial brushings in asthma patients compared with controls (Fig. 1a). Using fluorescent in situ hybridization, we demonstrated that miR-30a-3p was mainly located in the cytoplasm of airway epithelial cells (Fig. 1b).

**Table 1:** Subjects characteristics

	Control subjects	Asthma patients	P value
Number	16	51	
Age, year	38.19 ± 6.48	39.22 ± 12.56	0.759
Sex, M:F (%F)	8:8 (50)	15:36 (70.1)	0.13
Body mass index	22.08 ± 2.49	22.09 ± 2.65	0.99
FEV_1_, % predicted	99.70 ± 8.85	80.27 ± 17.24	0.0003
Methacholine PD_20_, mg	2.505 ± 0	0.14 ± 0.24	< 0.0001
Sputum eosinophil, %	0.45 ± 0.55	16.01 ± 17.67	< 0.0001
Biopsy eosinophil, #/mm^2^	0.39 ± 1.28	13.40 ± 13.27	< 0.0001
Blood eosinophil, /μL	109.3 ± 27.35	391.2 ± 47.15	0.0021
FeNO, ppb	20.05 ± 13.51	92.43 ± 59.40	< 0.0001

Values are presented as mean ± SD

*FEV*_*1*_ forced expiratory volume in the first second. *PD*_*20*_ provocative dosage required to cause a 20% decline in FEV_1_. The minimal and maximal provocative dosages were 0.01 and 2.505 mg, respectively. *FeNO* fraction of exhaled nitric oxide

Airway eosinophilic inflammation is a key feature of type 2-high asthma [1]. We found that miR-30a-3p had a strong negative correlation with parameters reflecting airway eosinophilia including eosinophil in induced sputum (Fig. 1c) and bronchial biopsies (Fig. 1d), and fraction of exhaled nitric oxide (Fig. 1e) in asthma patients. Furthermore, by measuring the expression of three epithelial signature genes (*CLCA1*, *POSTN*, *SERPINB2*) for type 2 status in endobronchial brushings using quantitative PCR [31], and by combination of these measurements to calculate a three-gene-mean for each subject, we found that epithelial miR-30a-3p expression negatively correlated with three-gene-mean (Fig. 1f). Our data indicate that epithelial miR-30a-3p expression associates with the type 2 status in asthma. Thus, epithelial miR-30a-3p may play a role in airway eosinophilic inflammation in type 2-high asthma.

### *RUNX2* is a target of miR-30a-3p

**Fig. 2:**
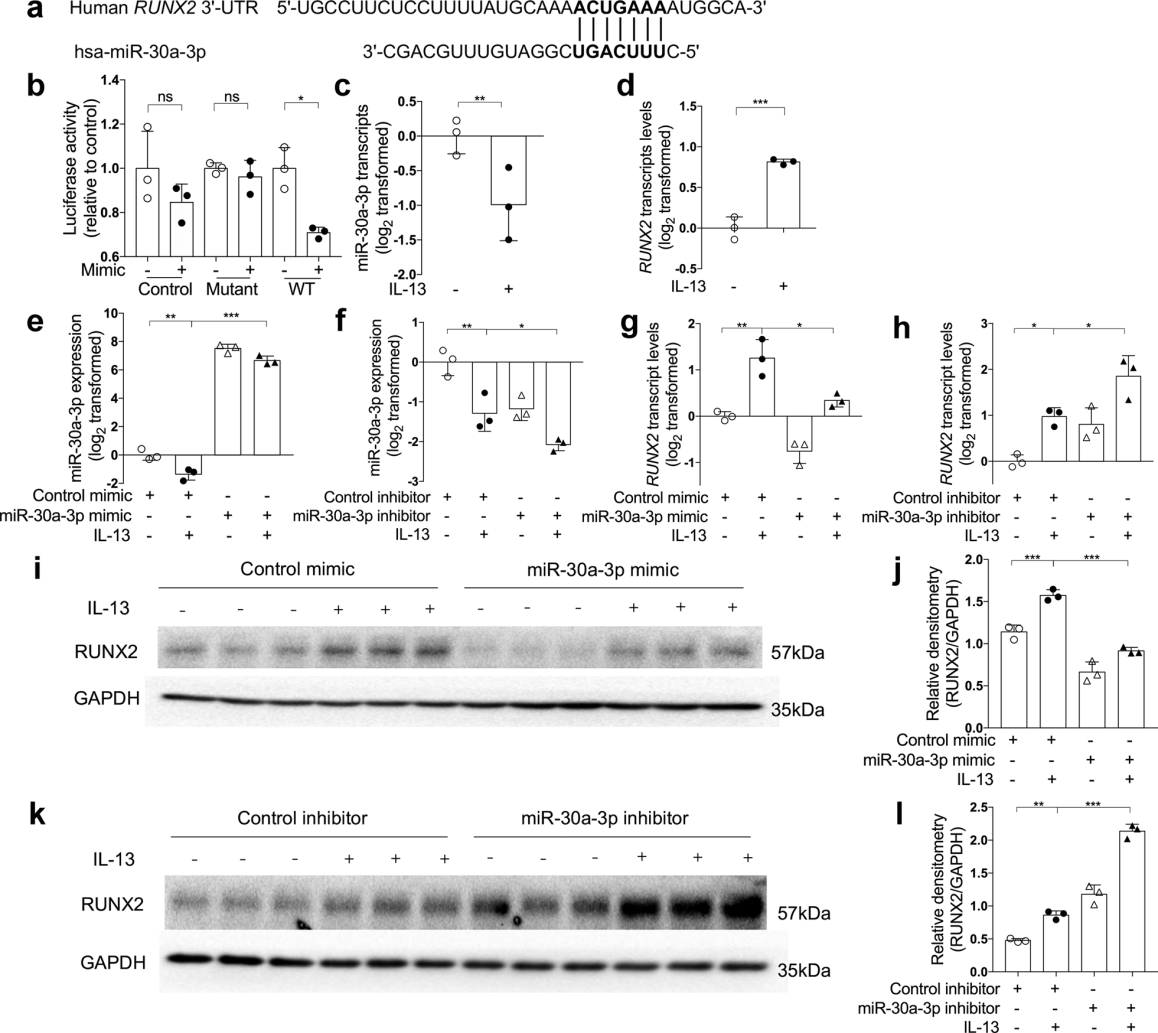
*RUNX2* is a target of miR-30a-3p. **a** The 3′‐UTR of *RUNX2* contains the region matching the seed sequence of hsa‐miR‐30a‐3p. **b** 3′-UTR luciferase reporter assay with vector harboring wild-type (WT), mutant *RUNX2* 3′-UTR or no 3′-UTR (control) co-transfected with miR-30a-3p mimic or control mimic, respectively. Luciferase activity was measured by dual-luciferase reporter assay system. The firefly luciferase activity was normalized to renilla luciferase activity. **c**, **d** The transcript levels of baseline and IL-13-induced miR-30a-3p (**c**) and *RUNX2* (**d**) in BEAS-2B cells were determined by quantitative PCR. The transcript levels were expressed as log2 transformed and relative to the mean of control group (two-tailed Student’s *t* test). **e**, **f** After control or miR-30a-3p mimic transfection with or without IL-13 stimulation, the transcript levels of miR-30a-3p were determined by quantitative PCR. **g**, **h** The transcript levels of *RUNX2*, after miR-30a-3p mimic (**g**) or inhibitor (**h**) transfection with or without IL-13 stimulation, were determined by quantitative PCR. The transcript levels were expressed as log2 transformed and relative to the mean of control group (one-way ANOVA followed by Tukey’s multiple comparison test). **i**–**l** The protein level of RUNX2 in BEAS-2B cells were determined by Western blotting after miR-30a-3p mimic (**i**, **j**) or inhibitor (**k**, **l**) transfection with or without IL-13 stimulation. Densitometry assay of the Western blotting results was analyzed using ImageJ, and the protein levels of RUNX2 were indexed to GAPDH. n = 3 wells per group. Data are mean ± SD. *P < 0.05; **P < 0.01; ***P < 0.001 (one-way ANOVA followed by Tukey’s multiple comparison test)

We predicted the candidate target genes of miR-30a-3p by using online algorithms including miRanda and TargetScan. *RUNX2* is one of the candidate target genes predicted by both online algorithms (Additional file 1: Table S2). The seed sequence of miR-30a-3p matches the 3′-untranslated region (UTR) of *RUNX2* (Fig. 2a). Co-transfection with the vector harboring wild-type *RUNX2* 3′-UTR and miR-30a-3p mimic decreased the luciferase activity. However, co-transfection with the vector containing mutant 3′-UTR or empty vector and miR-30a-3p mimic had no effect on the luciferase activity (Fig. 2b). This indicates that miR-30a-3p can directly bind to the 3′-UTR of *RUNX2*.

We used IL-13, a type 2 cytokine, to stimulate BEAS-2B bronchial epithelial cells. IL-13 decreased miR-30a-3p expression, but increased *RUNX2* expression in BEAS-2B cells (Fig. 2c, d). When transfection with miR-30a-3p mimic, IL-13-induced *RUNX2* mRNA and protein expression were significantly decreased (Fig. 2g, i, j). In contrast, IL-13-induced *RUNX2* mRNA and protein expression were further enhanced when transfection with miR-30a-3p inhibitor (Fig. 2h, k, l). Our data suggest that *RUNX2* is a target of miR-30a-3p.

### Epithelial RUNX2 is up‐regulated and correlates with airway eosinophilia in asthma

**Fig. 3:**
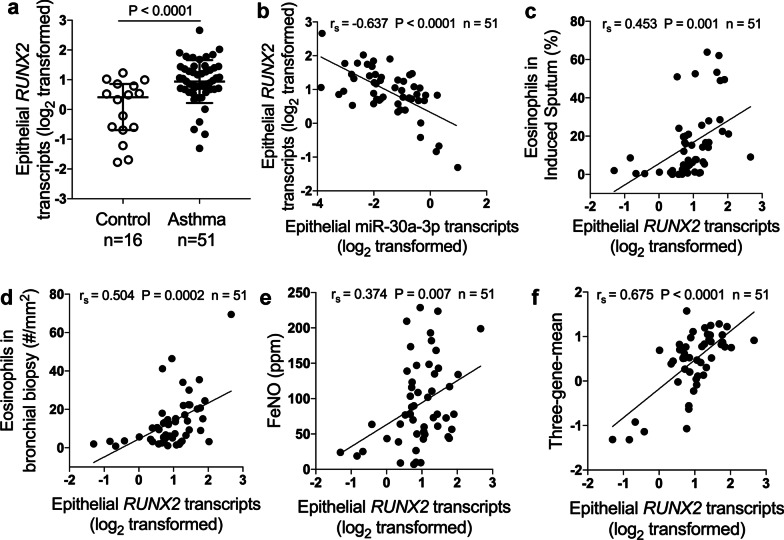
Epithelial RUNX2 is up‐regulated and correlates with airway eosinophilia. **a** Bronchial brushings from asthma patients (n = 51) and control subjects (n = 16) were subjected to quantitative PCR assays for *RUNX2* transcript levels. The transcript levels were expressed as log2 transformed and relative to the median value for controls (two‐tailed Mann–Whitney test). **b** Correlation assays between epithelial transcript levels of hsa-miR‐30a-3p and *RUNX2* in asthma patients (n = 51). **c**–**f** Correlation assays between epithelial *RUNX2* transcript levels and eosinophils in induced sputum (**c**) and bronchial biopsies (**d**), FeNO (**e**) and three‐gene‐mean of *CLCA1*, *POSTN* and *SERPINB2* (**f**) in asthma patients (n = 51). Correlation assays were performed using Spearman’s rank‐order correlation

We found that *RUNX2* transcript levels were significantly increased in bronchial brushings from asthma patients when compared with controls (Fig. 3a). In support of *RUNX2* as a target of miR‐30a‐3p, epithelial *RUXN2* transcripts negatively correlated with miR‐30a‐3p expression in asthma patients (Fig. 3b). We next analyze the correlation between epithelial *RUNX2* expression and airway eosinophilia. Epithelial *RUNX2* transcript levels positively correlated with eosinophils in induced sputum (Fig. 3c) and bronchial biopsies (Fig. 3d), FeNO (Fig. 3e), and the three‐gene‐mean (Fig. 3f). This indicates that RUNX2 may be involved in the pathogenesis of airway eosinophilic inflammation.

### RUNX2 binds to the promoter of *HMGB1*

**Fig. 4:**
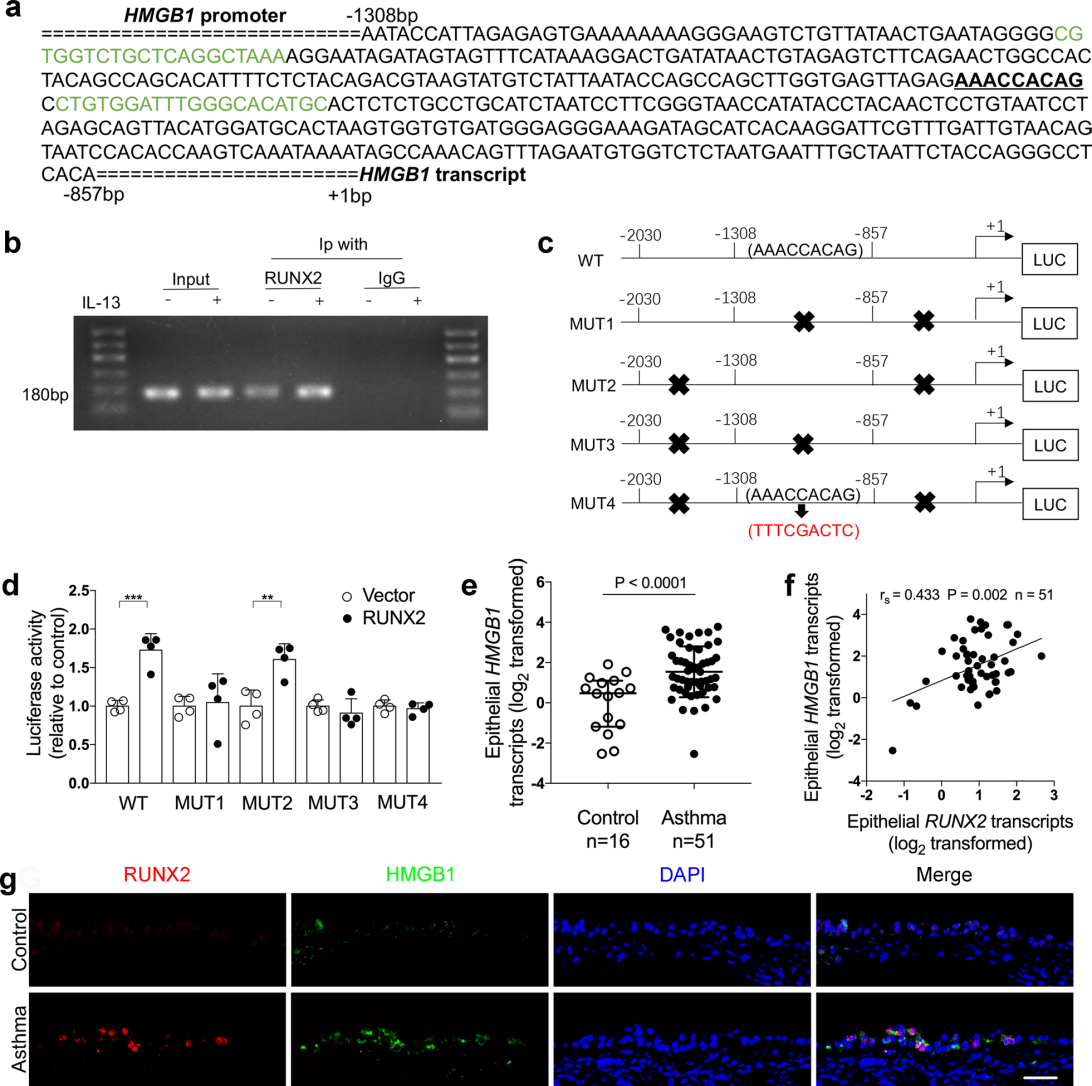
RUNX2 binds to the promoter of *HMGB1*. **a** The promoter region of *HMGB1* has a putative binding site for RUNX2. Sequence scheme of HMGB1 promoter region with the putative RUNX2 binding element underlined (AAACCACAG). Sequences marked in green are primers for ChIP-PCR assay in panel B. **b** ChIP-PCR assays to amplify the 180-bp region of HMGB1 promoter were performed to show direct binding of RUNX2 to *HMGB1* promoter in BEAS-2B cells. **c** Schematic presentation showing the luciferase reporter plasmid containing the wild type (WT), truncated, or mutant *HMGB1* promoter (MUT1–MUT4). MUT1 retains − 2030 to − 1308 bp, MUT2 retains − 1308 to − 857 bp, MUT3 retains − 857 to + 1 bp. MUT4 retains − 1308 to − 857 bp and the putative RUNX2 binding element AAACCACAG was replaced with TTTCGACTC. **d** The luciferase reporter plasmids containing WT and mutant *HMGB1* promoter were co-transfected with empty or RUNX2 cDNA expression vector. Luciferase activity was measured by dual-luciferase reporter assay system. The renilla luciferase activity was normalized to firefly luciferase activity (one-way ANOVA followed by Tukey’s multiple comparison test). n = 4 wells per group. Data are mean ± SD. *P < 0.05; **P < 0.01; ***P < 0.001. **e** *HMGB1* mRNA levels in bronchial brushings from asthma patients (n = 51) and controls (n = 16) were determined by quantitative PCR assays. The mRNA levels were expressed as log2 transformed and relative to the median value of controls (two‐tailed Mann–Whitney test). **f** Correlation assays between epithelial transcript levels of *RUNX2* and *HMGB1* in asthma patients (n = 51). Correlation assays were performed using Spearman's rank‐order correlation. **g** Representative images of RUNX2 (red) and HMGB1 (green) immunofluorescence staining in bronchial biopsies from controls and asthma patients. Nuclei were stained with DAPI (blue). Scale bar, 50 μm

HMGB1 plays a key role in the pathogenesis of airway inflammation in asthma [26]. According to a previous ChIP-on-chip analysis of RUNX2 [21], RUNX2 may bind to *HMGB1* gene. We predict the binding site of RUNX2 for *HMGB1* gene using JASPR (http://jaspar.genereg.net/) and found that *HMGB1* promoter DNA sequence, − 1308 to − 857 bp upstream of the *HMGB1* transcription start site, contains a 9-bp putative binding sequence for RUNX2 (Fig. 4a). Using ChIP-PCR analysis, we confirmed that RUNX2 directly binds to *HMGB1* promoter. Moreover, IL-13 treatment significantly enhanced the interaction between RUNX2 and HMGB1 compared to control cells (Fig. 4b).

To explore whether there are multiple binding sites for RUNX2 in the promoter region of *HMGB1*, luciferase reporters containing the wild type, truncated, or mutant *HMGB1* promoters were constructed (Fig. 4c). The luciferase activity of the reporter containing wild type *HMGB1* promoter was increased after co-transfection with RUNX2 cDNA plasmid. Of the four luciferase reporters containing mutant *HMGB1* promoter, luciferase activity was only increased in the mutant HMGB1 promoter containing the 9-bp binding sequence (− 1098 to − 1106 bp). Furthermore, mutation of the 9-bp binding sequence is sufficient to abrogate the increase of luciferase activity (Fig. 4d). This suggests that the *HMGB1* promoter regions contains a binding site for RUNX2.

### RUNX2 promotes HMGB1 expression in airway epithelial cells

We next investigate whether RUNX2 promotes HMGB1 expression in airway epithelial cells. *HMGB1* transcript levels in bronchial brushings were significantly up-regulated in asthma patients compared to control subjects (Fig. 4e). Moreover, epithelial *RUNX2* transcript levels in bronchial brushings were positively correlated with *HMGB1* transcript levels in asthma patients (Fig. 4f). Immunofluorescent staining of bronchial biopsies revealed that the expression of RUNX2 and HMGB1 were both increased in the epithelial cells of asthma patients compared to controls (Fig. 4g). These data from asthma patients suggest that RUNX2 may contribute to HMGB1 expression in airway epithelial cells.

**Fig. 5:**
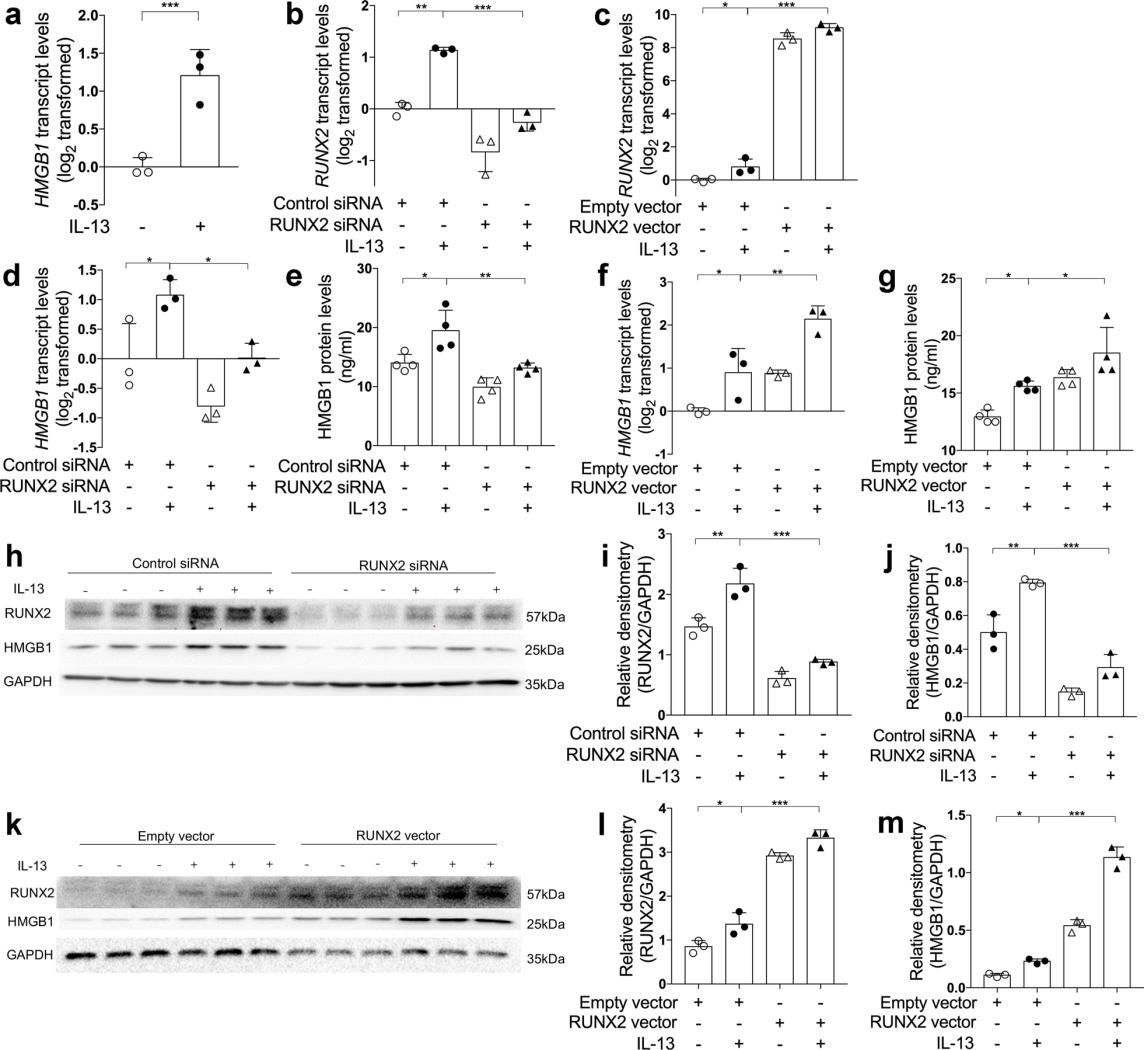
RUNX2 promotes HMGB1 expression in airway epithelial cells. **a** The transcript levels of miR-30a-3p in control and IL-13-stimulated BEAS-2B cells were determined by quantitative PCR. The transcript levels were expressed as log2 transformed and relative to the mean of control group (two-tailed Student’s *t* test). **b**–**d**, **f** The transcript levels of *RUNX2* (**b**, **c**) and *HMGB1* (**d**, **f**) after transfection with control or RUNX2 siRNA with or without IL‐13 stimulation, and empty or RUNX2 cDNA expression vector with or without IL‐13 stimulation were detected by quantitative PCR. The transcript levels were expressed as log2 transformed and relative to the mean of control group. **e**, **g** The protein levels of HMGB1 in cell culture media were determined by ELISA, after transfection with control or RUNX2 siRNA (**e**) with or without IL‐13 stimulation, and empty or RUNX2 cDNA expression vector (**g**) with or without IL‐13 stimulation. **h**–**m** The protein levels of RUNX2 and HMGB1 in BEAS-2B were determined by Western blotting after transfection with control or RUNX2 siRNA with or without IL‐13 stimulation (**h**–**j**), and empty or RUNX2 cDNA expression vector with or without IL‐13 stimulation (**k**–**m**)*.* Densitometry assay of the Western blotting results was analyzed using ImageJ, and the protein levels of RUNX2 and HMGB1 were indexed to GAPDH. n = 3–4 wells per group. Data are mean ± SD. *P < 0.05; **P < 0.01; ***P < 0.001 (one-way ANOVA followed by Tukey’s multiple comparison test)

We further examined HMGB1 expression after RUNX2 knockdown or overexpression in BEAS-2B cells. Similar to *RUNX2*, the expression of *HMGB1* was increased after IL-13 stimulation (Fig. 5a). RUNX2 knockdown by transfecting with siRNA against RUNX2 decreased IL-13-induced HMGB1 expression in BEAS-2B cells compared to the cells transfected with non-targeting control siRNA (Fig. 5b, d, e, h–j). In contrast, RUNX2 overexpression by transfecting with a RUNX2 cDNA expression vector enhanced IL-13-induced HMGB1 expression compared with the cells transfected with empty vector (Fig. 5c, f, g, k–m). Our data indicate that RUNX2 promotes the expression of HMGB1 in airway epithelial cells.

In addition, we found that miR-30a-3p transcripts levels negatively correlated with *HMGB1* transcripts in bronchial brushings from asthma patients (Additional file 1: Fig. S1a). In BEAS-2B cells, miR-30a-3p overexpression by transfecting with miR-30a-3p mimic suppressed baseline and IL-13-induced *HMGB1* mRNA and protein expression. In contrast, transfection with miR-30a-3p inhibitor enhanced IL-13-induced HMGB1 expression (Additional file 1: Fig. S1b–e). Together, our data suggest miR-30a-3p inhibits HMGB1 expression by targeting RUNX2.

### Airway overexpression of mmu-miR-30a-3p suppresses airway eosinophilia in a murine model of allergic airway inflammation

**Fig. 6:**
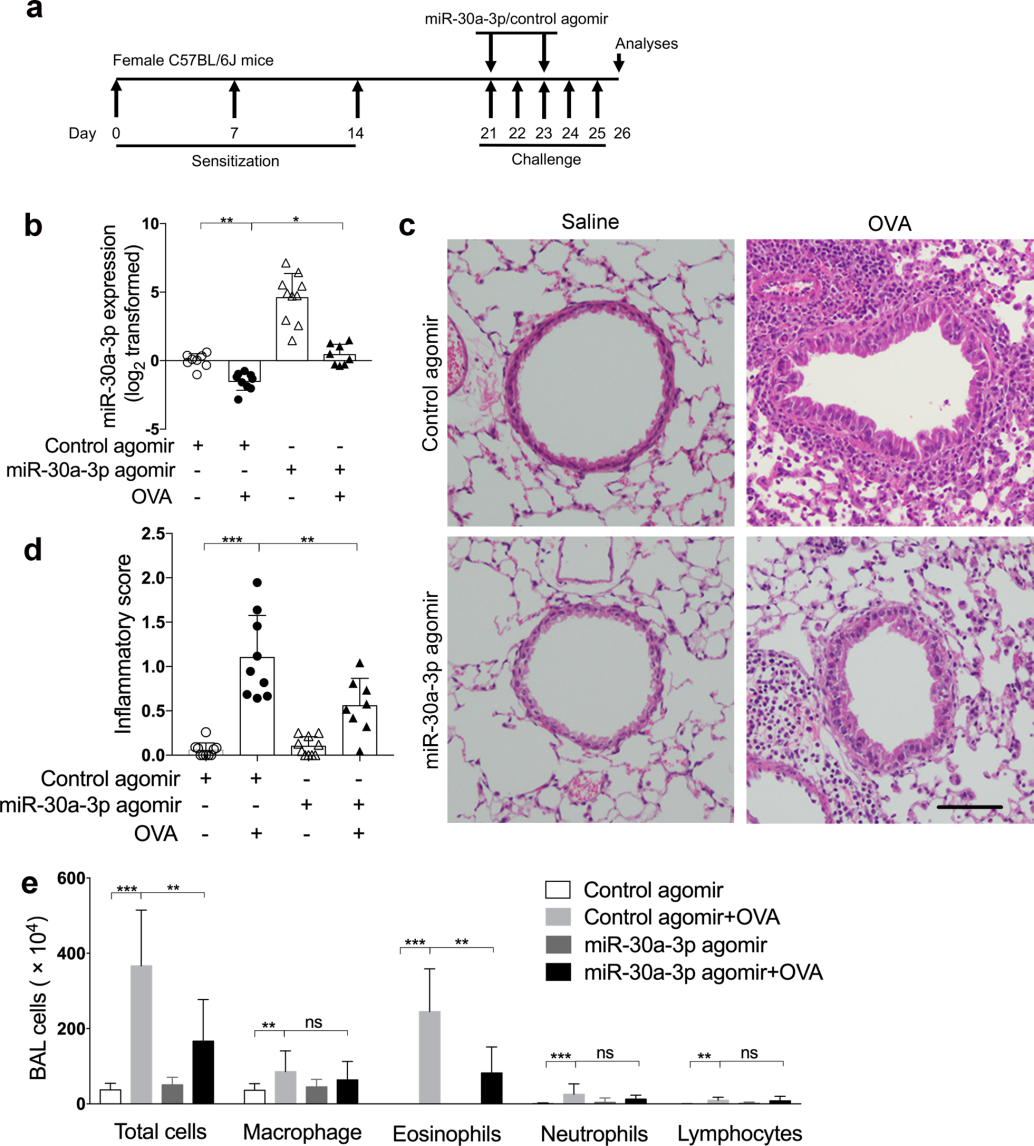
MiR-30a-3p overexpression suppresses airway eosinophilic inflammation in a mouse model of allergic airway inflammation. **a** Experimental schedule. mmu‐miR‐30a‐3p or control agomir was administered intranasally 2 h before OVA challenge on days 21 and 23. **b** The transcript levels of miR-30a-3p in mouse lungs were determined by quantitative PCR. The transcript levels were expressed as log2 transformed and relative to the mean of control group. **c** Representative H&E staining of mouse lung sections. Scale bar, 50 μm. **d** Lung inflammatory scores were calculated as described in ‘Materials and methods’. **e** Counts for macrophages, eosinophils, lymphocytes and neutrophils in BAL fluid. n = 7–10 mice per group. Data are mean ± SD. *P < 0.05; **P < 0.01; ***P < 0.001 (one-way ANOVA followed by Tukey’s multiple comparison test)

We further explored the role of miR‐30a‐3p in asthma using a murine model of allergic airway inflammation. Mice were sensitized and challenged with OVA, mmu‐miR‐30a‐3p or control agomir was administered intranasally 2 h before OVA challenge on days 21, 23 (Fig. 6a). Compared with control mice, we found that the expression of mmu-miR-30a-3p was decreased in OVA-challenged mice using quantitative PCR and fluorescence in situ hybridization. MiR-30a-3p agomir administration significantly increased airway miR-30a expression (Fig. 6b, Additional file 1: Fig. S2). OVA sensitization and challenge resulted in infiltration of inflammatory cells around the airways as assessed using H&E staining and airway inflammation scoring (Fig. 6c, d). However, mmu-miR-30a-3p overexpression suppressed airway inflammation in OVA-challenged mice (Fig. 6c, d). Moreover, BALF cell counting revealed that mmu-miR-30a-3p overexpression decreased the number of eosinophils in BALF from OVA-challenged mice (Fig. 6e). This suggests that miR-30a-3p protects against airway eosinophilia in mouse asthma model. The transcript levels of *Ccl11* and *Ccl24* were both increased in lung tissue of OVA-challenged mice, but airway overexpression of miR-30a-3p did not suppress *Ccl11* and *Ccl24* expression (Additional file 1: Fig. S3). This indicates that miR-30a-3p may not affect the expression of eosinophil specific chemokines.

**Fig. 7:**
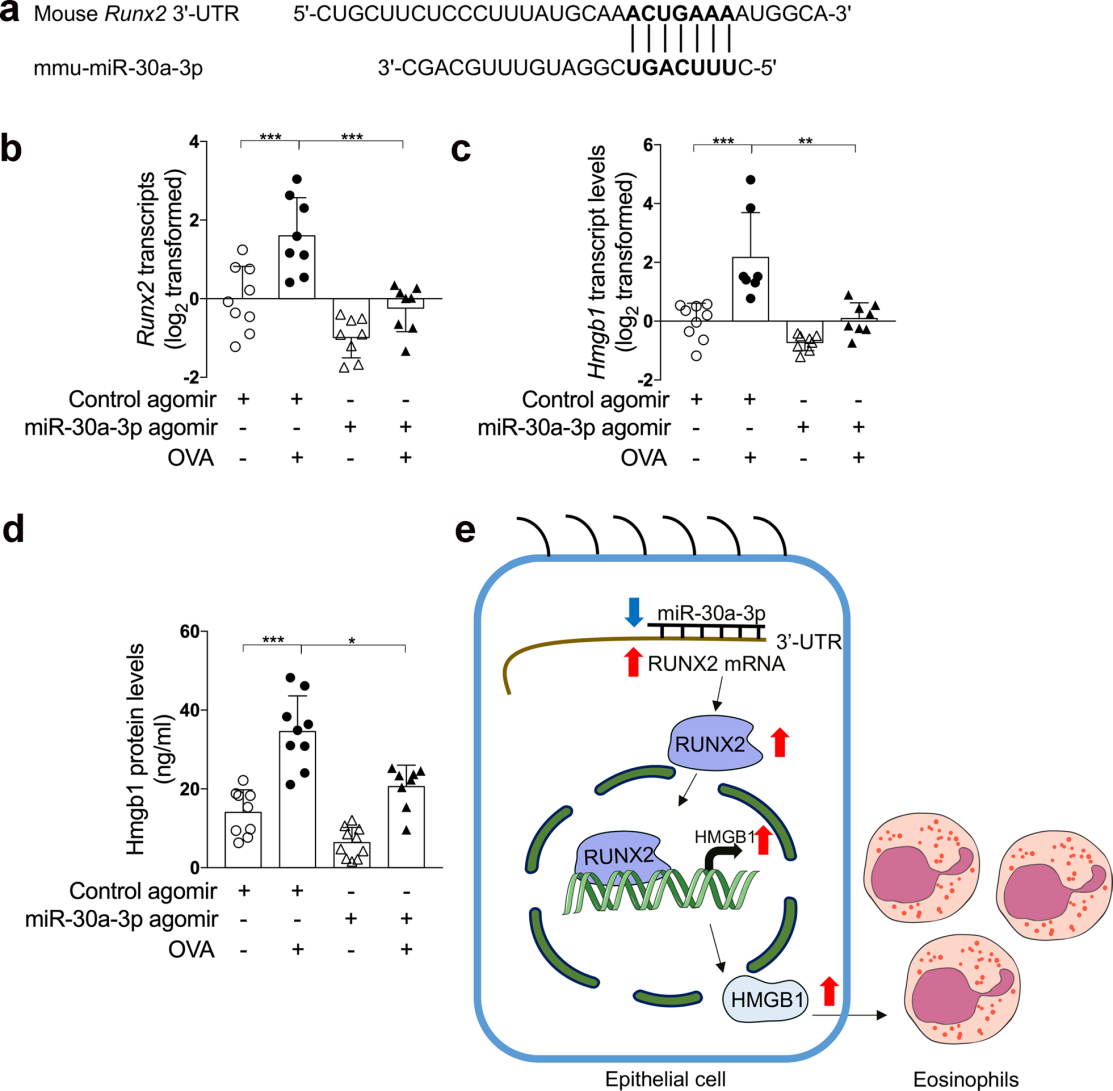
mmu‐miR‐30a‐3p overexpression suppresses Runx2 and Hmgb1 up‐regulation in a mouse model of allergic airway inflammation. **a** The 3′‐UTR of *Runx2* contains the region matching the seed sequence of mmu‐miR‐30a‐3p. **b**, **c** The transcript levels of *Runx2* (**b**) and *Hmgb1* (**c**) in mouse lungs were determined by quantitative PCR. The transcript levels were expressed as log2 transformed and relative to the mean of control group. **d** The protein levels of Hmgb1 in BALF were determined by ELISA. n = 7–10 mice per group. Data are mean ± SD. *P < 0.05; **P < 0.01; ***P < 0.001 (one-way ANOVA followed by Tukey’s multiple comparison test). **e** A model of epithelial microRNA-30a-3p targeting RUNX2/HMGB1 axis to suppress airway eosinophilic inflammation in asthma

MiR‐30a‐3p is conserved across species, and the seed sequence of mmu-miR-30a-3p matches the 3′-UTR of *Runx2* (Fig. 7a). Using quantitative PCR and ELISA, we found that expression of Runx2 and Hmgb1 was markedly increased in OVA-challenge mice compared with control mice. However, airway overexpression of miR-30a-3p suppressed Runx2 and Hmgb1 expression (Fig. 7b–d). This supports our in vitro findings that miR-30a-3p targets RUNX2, and RUNX2 promotes HMGB1 expression. In addition, miR-30a-3p overexpression decreased the number of mucus cells and Muc5ac mRNA expression in OVA-challenged mice as assessed by PAS staining and quantitative PCR, respectively (Additional file 1: Fig. S4). Since RUNX2 was reported to regulate the differentiation of goblet cells, our data suggests that miR-30a-3p may suppress mucus overproduction by targeting RUNX2.

## Discussion

In the present study, we reported that miR‐30a‐3p expression was downregulated in bronchial epithelium in treatment‐naïve asthma patients, in IL‐13‐stimulated human bronchial epithelial cells, and in the airways of a murine model of allergic airway inflammation. Moreover, epithelial miR-30a-3p expression was significantly correlated with airway eosinophilia in asthma patients. Our findings are consistent with the report that miR-30a-3p expression was decreased in the peripheral blood of asthmatic patients [15].

MiRNAs regulate gene expression by binding to the seed sequence in the 3′-UTR of target gene. We verified that *RUNX2* is a target gene of miR-30a-3p. RUNX2 expression was significantly increased in bronchial epithelium of asthma patients, IL‐13‐stimulated human bronchial epithelial cells and in OVA‐challenged mice airways. It was reported that upregulation of RUNX2 in vascular smooth muscle cells increases the expression of inflammatory cytokines and promotes infiltration of inflammatory cells [32]. Consistently, we found that epithelial *RUXN2* expression was positively correlated with airway eosinophilia in asthma. This suggests that miR‐30a-3p may play a role in airway eosinophilia by targeting *RUNX2*.

RUNX2 is a transcription factor and a previous report suggests that RUNX2 may interact with HMGB1 [21]. However, there was no reports providing direct evidence that RUNX2 binds to *HMGB1* promoter and regulates its expression. In this study, we found that RUNX2 directly binds to a 9-bp sequence in the promoter of *HMGB1* gene. IL-13 enhances the binding of RUNX2 to the HMGB1 promoter. In BEAS-2B cells, we showed that knockdown or overexpression of RUNX2 inhibited or intensified IL-13-induced HMGB1 expression, respectively.

HMGB1 can interact with the receptors including RAGE, TLR2 and TLR4 [33, 34]. Activation of these receptors finally leads to the production of various pro-inflammatory cytokines [35, 36]. We demonstrated that, like RUNX2, HMGB1 expression was increased in bronchial epithelium of asthma patients, IL‐13‐stimulated human bronchial epithelial cells and OVA‐challenged mice airways. In support of the role miR-30a-3p/RUNX2 in the regulation of HMGB1 expression, epithelial HMGB1 expression was negatively correlated with epithelial miR‐30a‐3p expression, and positively correlated with epithelial RUNX2 expression.

RUNX2 can also bind to the promoter of secreted phosphoprotein 1 (SPP1) [37]. SPP1, also named as osteopontin, is reported to be involved in airway inflammation [12, 38]. Thus, RUNX2 may contribute to airway inflammation through regulating Spp1 expression in addition to promoting HMGB1 expression. Interestingly, RUNX2 also regulates the expression of SPDEF, a critical factor for goblet cell differentiation [20].

Furthermore, we demonstrated the airway overexpression of mmu-miR-30a-3p suppressed airway inflammation in OVA-challenged mice. The sequence of mmu-miR-30a-3p is identical to hsa-miR-30a-3p, and the 3′-UTR of murine *Runx2* gene has binding sites for the seed sequence of miR-30a-3p. In support of *Runx2* as a target of miR-30a-3p and as a transcription factor to regulate *Hmgb1* expression, mmu-miR-30a-3p overexpression decreased Runx2 and Hmgb1 expression in OVA-challenged mice. Additionally, mmu-miR-30a-3p overexpression also decreased the number of mucus cells and the expression of Muc5ac. This is consistent with the report that RUNX2 can regulate the differentiation of goblet cells [20].

Our study has several limitations. First, the disproportionate sample size of asthma patients (n = 51) subjects and control subjects (n = 16) may lead to analysis bias. We repeated the statistical analyses in 16 asthma patients randomly selected from the cohort of 51 asthma patients in comparison to the 16 control subjects, and the results were consistent with those shown in Fig. 1 (Additional file 1: Fig. S5). This suggests there was no significant bias. Second, because a miRNA can target multiple genes expression, we can’t exclude the possibility that miR-30a-3p regulates airway inflammation by targeting genes other than *RUNX2*.

## Conclusion

We conclude that epithelial miR-30a-3p could possibly target RUNX2/HMGB1 axis to suppress airway eosinophilic inflammation in asthma.

## Supplementary Information

**Additional file 1: Table S1.** Primers for quantitative PCR. **Table S2.** Candidate targets of miR-30a-3p predicted by online algorithms miRanda and TargetScan. **Figure S1.** Inhibition of miR-30a-3p enhances the expression of HMGB1. **Figure S2.** Mmu-miR-30a-3p agomir is sufficient to abrogate the decrease of the expression of mmu-miR-30a-3p in OVA-challenge mice. **Figure S3.** Mmu-miR-30a-3p overexpression did not affect Ccl11 and Ccl24 expression in mouse lung tissue. **Figure S4.** Mmu-miR-30a-3p overexpression decreased the mucus production in OVA sensitized and challenged mice. **Figure S5.** The expression of miR-30a-3p, RUNX2, HMGB1, and the correlation between miR-30a-3p and airway eosinophilia in 16 asthma patients randomly selected from the cohort of 51 asthma patients.

## Data Availability

All data generated or analyzed during this study are included in this published article and its Additional file 1.

## Abbreviations

ChIP: Chromatin immunoprecipitation
RUNX2: RUNX family transcription factor 2
HMGB1: High mobility group box 1
AHR: Airway hyperresponsiveness
IL: Interleukin
CCL: C–C motif chemokine ligand
miRNA: MicroRNA
SPDEF: SAM pointed domain containing ETS transcription factor
TLR: Toll-like receptor
RAGE: Receptor for advanced glycation end products
PD_20_: Dosage of methacholine provoking a 20% fall
FEV_1_: Forced expiratory volume in the first second
FeNO: Fraction of exhaled nitric oxide
BALF: Bronchoalveolar lavage fluid
H&E: Hematoxylin and eosin
PAS: Periodic acid-Schiff
PVDF: Polyvinylidene difluoride
ELISA: Enzyme linked immunosorbent assay
SD: Standard deviation
