# Supplementary material for: Epithelial microRNA-30a-3p targets RUNX2/HMGB1 axis to suppress airway eosinophilic inflammation in asthma
Source: Respir Res. 2022 Jan 29;23:17. doi: 10.1186/s12931-022-01933-x (PMC8800331; doi:10.1186/s12931-022-01933-x)
Supplement: Supplementary file 1 — Additional file 1: Table S1. Primers for quantitative PCR. Table S2. Candidate targets of miR-30a-3p predicted by online algorithms miRanda and TargetScan. Figure S1. Inhibition of miR-30a-3p enhances the expression of HMGB1. Figure S2. Mmu-miR-30a-3p agomir is sufficient to abrogate the decrease of the expression of mmu-miR-30a-3p in OVA-challenge mice. Figure S3. Mmu-miR-30a-3p overexpression did not affect Ccl11 and Ccl24 expression in mouse lung tissue. Figure S4. Mmu-miR-30a-3p overexpression decreased the mucus production in OVA sensitized and challenged mice. Figure S5. The expression of miR-30a-3p, RUNX2, HMGB1, and the correlation between miR-30a-3p and airway eosinophilia in 16 asthma patients randomly selected from the cohort of 51 asthma patients. [file 12931_2022_1933_MOESM1_ESM.pdf]

Additional File 1:

Epithelial microRNA-30a-3p targets RUNX2/HMGB1 axis to suppress airway eosinophilia in asthma

Wenliang Wu[1,2], Jiali Gao[1,2], Dian Chen[1,2], Gongqi Chen[1,2], Yuchen Feng[1,2], Chenli Chang[1,2], Shengchong Chen[1,2], Lingling Yi[1,2]†, Guohua Zhen[1,2]†

[1]Division of Respiratory and Critical Care Medicine, Department of Internal Medicine, Tongji Hospital, Tongji Medical College, Huazhong University of Science and Technology, Wuhan, China; [2]Key Laboratory of Respiratory Diseases, National Health Commission of People's Republic of China, and National Clinical Research Center for Respiratory Diseases, Wuhan, China

ADDITIONAL TABLES

Table S1. Primers for quantitative PCR

**Gene**	**Species**		**Sequence**
GUSB	Human	Forward	GTCTGCGGCATTTTGTCGG
		Reverse	CACACGATGGCATAGGAATGG
RUNX2	Human	Forward	TGGTTACTGTCATGGCGGGTA
		Reverse	TCTCAGATCGTTGAACCTTGCTA
HMGB1	Human	Forward	TGTGCCTCGCTGAGGAAAAAT
		Reverse	GGGTGCATTGGGATCCTTGA
CLCA1	Human	Forward	ATGGCTATGAAGGCATTGTCG
		Reverse	TGGCACATTGGGGTCGATTG
POSTN	Human	Forward	GACCGTGTGCTTACACAAATTG
		Reverse	AAGTGACCGTCTCTTCCAAGG
SERPINB2	Human	Forward	TCCTGGGTCAAGACTCAAACC
		Reverse	CATCCTGGTATCCCCATCTACA
β-actin	Mouse	Forward	GTGACGTTGACATCCGTAAAGA
		Reverse	GCCGGACTCATCGTACTCC
Runx2	Mouse	Forward	GACTGTGGTTACCGTCATGGC
		Reverse	ACTTGGTTTTTCATAACAGCGGA
Hmgb1	Mouse	Forward	TGGCTTTTGTCCCTCATCCTT
		Reverse	AGAGGCCGCAGTTTCCTATC
Mu5ac	Mouse	Forward	CAGGACTCTCTGAAATCGTACCA
		Reverse	GAAGGCTCGTACCACAGGG

Table S2. Candidate targets of miR-30a-3p predicted by online algorithms miRanda and TargetScan

Gene symbol	Gene ID
CDC73	79577
ZEB2	9839
PCLO	27445
NBEAL1	65065
PANK3	79646
CREB1	1385
SLC12A6	9990
RUNX2	860
PRKAA2	5563
TBC1D23	55773
RGS7BP	401190
MED12L	116931
ROR1	4919
NEGR1	257194
UBE2G1	7326
RALA	5898
STAU1	6780
UBE2J1	51465
IGF1	3479
COLEC12	81035
FAM20B	9917
PAIP2	51247
SLC36A4	120103
CCDC6	8030
GPR158	57512
SLC25A33	84275
ZNHIT6	54680
MRPL30	51263
HIRA	7290
SEMA3C	10512
TRMT10B	158234
PMEL	6490
SLITRK6	84189
LSS	4047
WDR44	54521
PYROXD1	79912
MEOX2	4223
GPRASP2	114928
KIAA1324L	222223
SYT4	6860

GPR176	11245
UFSP2	55325
RANBP3L	202151
GOSR1	9527
KANSL1L	151050
STIM2	57620
MAP3K2	10746
PHLDA1	22822
NOD1	10392
ROBO1	6091
EGR1	1958
SNRK	54861
PCSK5	5125
RYR3	6263
GPR180	160897
ZNF827	152485
STK38L	23012
TCP11L1	55346
XPNPEP3	63929
RFK	55312
SYT14	255928
CHURC1	91612
RB1	5925
POU4F1	5457
COL12A1	1303
SLC5A3	6526
RIF1	55183
MEI4	101928601
SH3GLB1	51100
FXR1	8087
HMGN4	10473
MTHFD2	10797
ELK3	2004
FGF7	2252
EPAS1	2034
TXLNB	167838
ATP11C	286410
FAM98A	25940
RTP4	64108
CEP152	22995
TOMM20	9804
USP1	7398
CACYBP	27101
TUSC3	7991

NPHP3	27031
CENPL	91687
NAPG	8774
CHMP1B	57132
UBXN2B	137886
FAM104A	84923
CCDC112	153733
TRIM72	493829
SLC35F3	148641
HOXB7	3217
ZNF430	80264
TMEM47	83604
CNPY2	10330

ADDITIONAL FIGURES

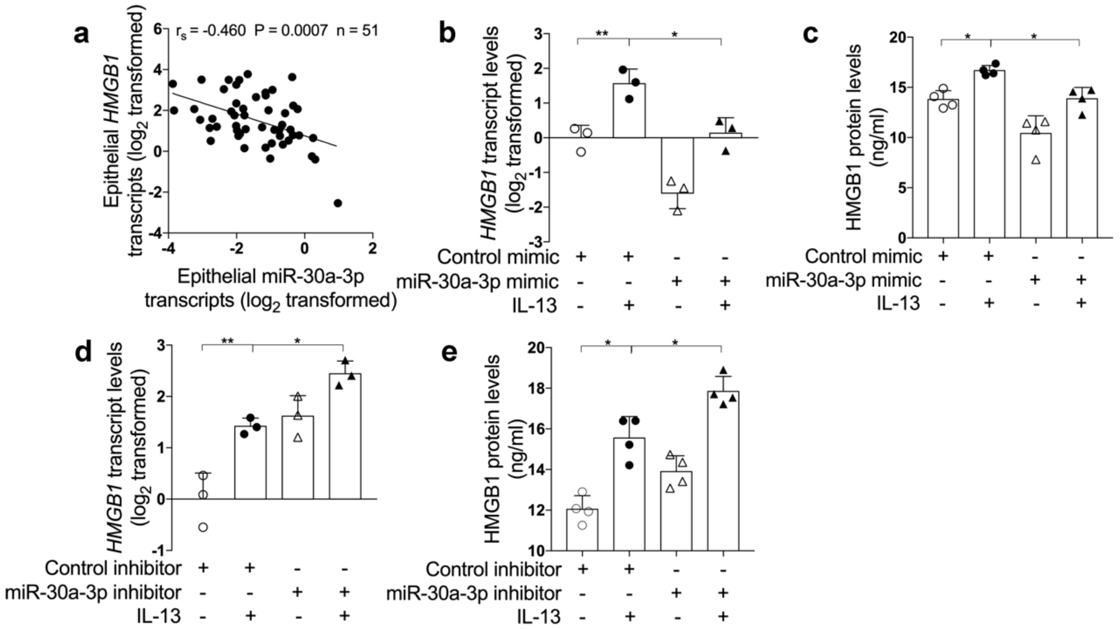

Figure S1. Inhibition of miR-30a-3p enhances the expression of HMGB1. (**a**) Correlation assays between epithelial transcript levels of has-miR-30a-3p and HMGB1 in asthma patients (n=51). Correlation assays were performed using Spearman's rank-order correlation. (**b**-**c**) After control or miR-30a-3p mimic transfection with or without IL-13 stimulation, the transcript levels of *HMGB1* (***b***) were determined by quantitative PCR, and the protein levels of HMGB1 (***c***) in cell culture media were determined by ELISA. (**d**-**e**) After control or miR-30a-3p inhibitor transfection with or without IL-13 stimulation, the transcript levels of *HMGB1* (***d***) were determined by quantitative PCR, and the protein levels of HMGB1 (***e***) in cell culture media were determined by ELISA. The transcript levels were expressed as log2 transformed and relative to the mean of control group. n = 3~4 wells per group. Data are mean ± SD. *P<0.05 (one-way ANOVA followed by Tukey's multiple comparison test). The data are representative of three independent experiments.

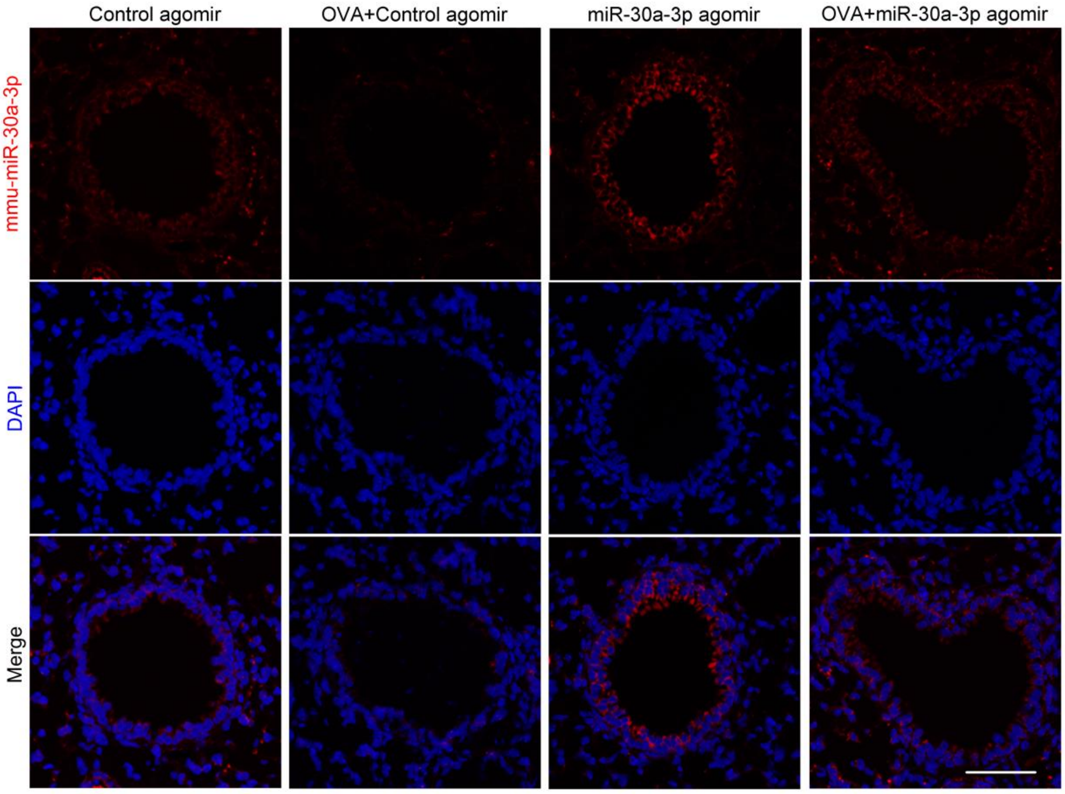

Figure S2. Mmu-miR-30a-3p agomir is sufficient to abrogate the decrease of the expression of mmu-miR-30a-3p in OVA-challenged mice. Representative images of fluorescence in situ hybridization of miR-30a-3p in mouse lung sections. Scale bar, 50 μm.

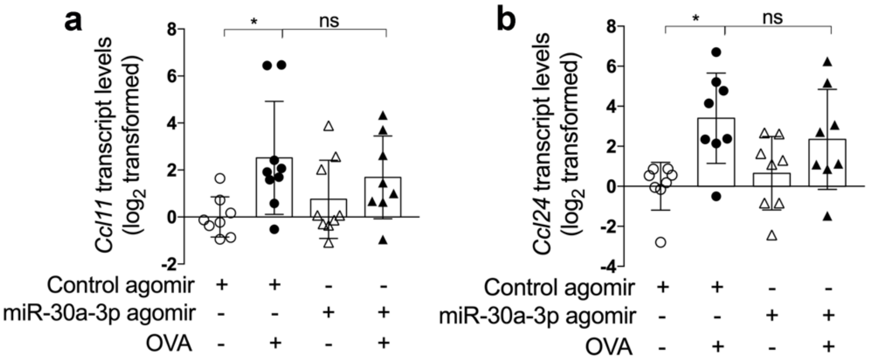

Figure S3. Mmu-miR-30a-3p overexpression did not affect Ccl11 and Ccl24 expression in mouse lung tissue. (**a**) The transcript levels of Ccl11 in mouse lungs were determined by quantitative PCR. (**b**) The transcript levels of Ccl24 in mouse lungs were determined by quantitative PCR. The transcript levels were expressed as log2 transformed and relative to the mean of control group. n = 7 - 9 mice per group. Data are mean ± SD. *P<0.05 (one-way ANOVA followed by Tukey's multiple comparison test).

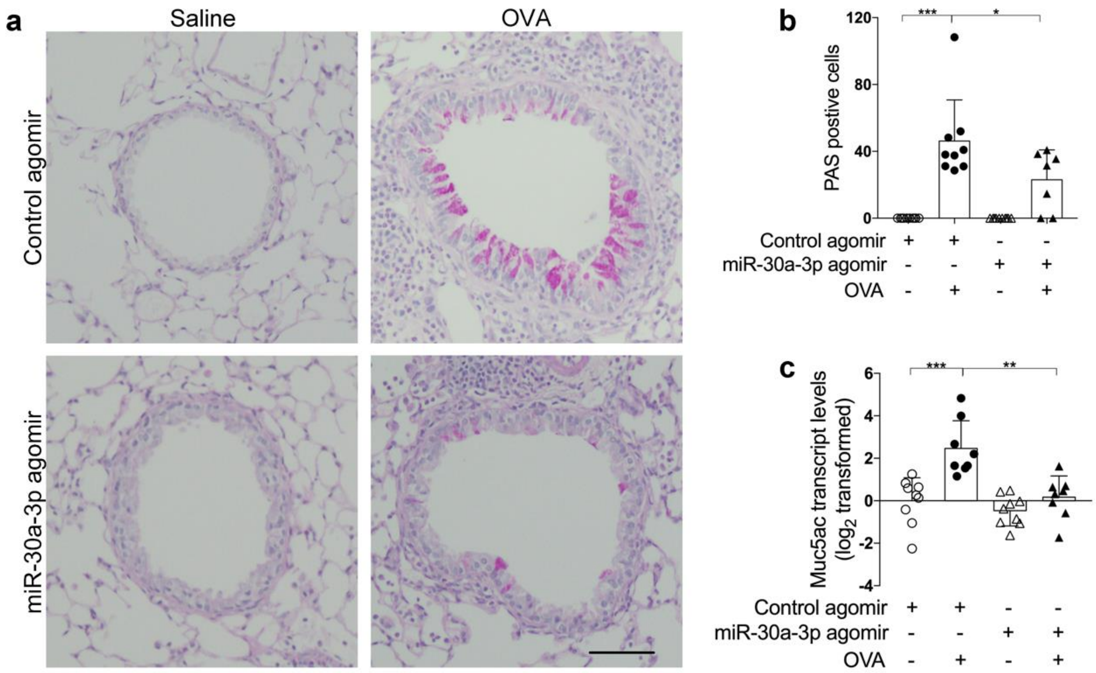

Figure S4. Mmu-miR-30a-3p overexpression decreased the mucus production in OVA sensitized and challenged mice. (**a**) PAS staining for mucus in representative lung sections. Scale bar, 50μm. (**b**) The numbers of PAS-staining-positive cells were counted in five random fields for each lung section at ×200 magnification. (**c**) *Muc5ac* transcripts levels in mouse lungs were determined by quantitative PCR. The transcript level was expressed as log2 transformed and relative to control. Data are mean ± SD. ***$P < 0.001$, **$P < 0.01$, *$P < 0.05$. One-way ANOVA followed by Tukey's multiple comparison test.

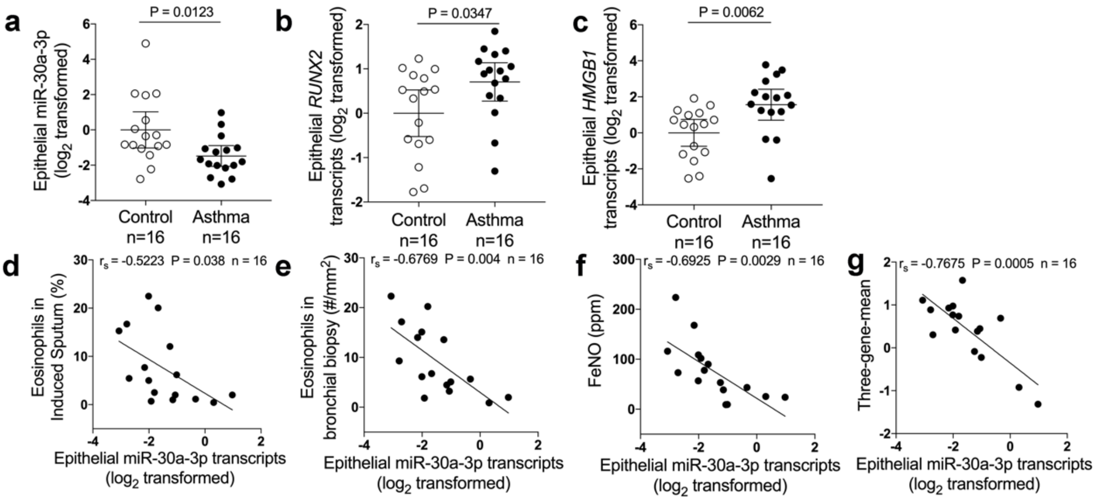

Figure S5. The expression of miR-30a-3p, RUNX2, HMGB1, and the correlation between miR-30a-3p and airway eosinophilia in 16 asthma patients randomly selected from the cohort of 51 asthma patients. The expression of miR-30a-3p, RUNX2, HMGB1, and the correlation between miR-30a-3p and airway eosinophilia were re-analyzed in 16 asthma patients randomly selected from the cohort of 51 asthma patients, to avoid the statistical analysis bias that could be caused by the disproportionate sample size of control (n = 16) and patients (n = 51). Fifty-one asthma patients were labeled (1-51) in the order of recruited time. Online algorithm (www.cnstat.org/randomization/simple-sampling) was used to get sixteen numbers randomly from one to fifty-one. The labeled patients with the same sixteen numbers were compared with controls. (**a-c**) Bronchial brushings from asthma patients (n=16) and control subjects (n=16) were subjected to quantitative PCR assays of hsa-miR-30a-3p, RUNX2 and HMGB1 transcript levels. The transcript levels were expressed as log2 transformed and relative to the median value for controls (two-tailed Mann–Whitney test). (**d-g**) Correlation assays between epithelial miR-30a-3p transcript levels and

eosinophils in induced sputum (**d**) and bronchial biopsies (**e**), FeNO (**f**), and three-gene-mean of CLCA1, POSTN and SERPINB2 (**g**) in asthma patients (n=16). Correlation assays were performed using Spearman's rank-order correlation.
